# Advanced Oxidation via Hydrodynamic Cavitation and Ozonation for Enhanced Decolorization and Biodegradability of Triazo Dyes in Textile Wastewater

**DOI:** 10.1021/acsomega.5c09276

**Published:** 2025-12-09

**Authors:** Rhayssa de Brito, Rodrigo B. Carneiro, Julio C. S. I. Gonçalves, Sávia Gavazza, Márcia H. R. Z. Damianovic

**Affiliations:** † Biological Processes Laboratory (LPB), São Carlos School of Engineering, 28133 University of São Paulo (USP), 1100, João Dagnone Ave., Santa Angelina, São Carlos, São Paulo 13563-120, Brazil; ‡ Laboratory of Chromatography (CROMA), São Carlos Institute of Chemistry, University of São Paulo (USP), 400, Trabalhador São-Carlense Ave., São Carlos, São Paulo 13566-590, Brazil; § Institute of Technological and Exact Sciences, Federal University of Triângulo Mineiro (UFTM), 1400, Randolfo Borges Júnior Ave., Uberaba, Minas Gerais 38064-200, Brazil; ∥ Laboratory of Environmental Sanitation, Department of Civil and Environmental Engineering, Federal University of Pernambuco (UFPE), Acadêmico Hélio Ramos Ave., Recife, Pernambuco 50740-530, Brazil

## Abstract

Textile wastewater containing azo dyes poses environmental and health risks if untreated. This study evaluated the decolorization of the azo dye Direct Blue 71 (DB71) using advanced oxidative processes (AOPs), specifically hydrodynamic cavitation (HC) and ozonation, both independently and in combination with biological treatments. HC as a pretreatment significantly improved DB71 biodegradability, achieving a 95.7% removal efficiency. Anaerobic batch tests showed that cavitation pretreatment doubled methane production (6.81 N mL-CH_4_) compared to untreated wastewater (2.96 N mL-CH_4_) and reduced dye removal time from 25 to 16 h. Thirteen central composite design experiments indicated organic carbon removal ranging from 7.6% to 58.3%, depending on the ozone flow rate (1.5–4.5 g-O_3_ h^–1^) and exposure time (30–100 min). Combining cavitation with 1 g-O_3_ h^–1^ fully removed DB71 and its byproducts in under 20 min, thanks to the synergistic effect of AOPs. These results demonstrate that combining HC with ozonation significantly enhances the oxidative degradation of recalcitrant azo dyes compared to individual processes. Moreover, the integration of AOPs with biological treatment provides a synergistic pathway that improves dye decolorization, increases biodegradability, and reduces the inhibitory effects of toxic intermediates, offering a potentially sustainable and promising strategy for textile wastewater remediation.

## Graphical abstract

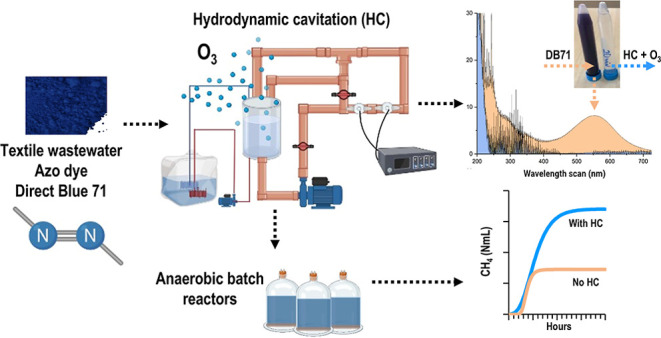

## 1 Introduction

Wastewaters produced by industrial dyeing processes are notably complex, characterized by high concentrations of inorganic salts, elevated organic loads, low biodegradability, and significant salinity.^1,2^ Azo dyes, particularly triazo dyes, are chemically stable and highly resistant to biodegradation, persisting in natural environments.^3^ In addition to textile finishing operations, which often generate high sulfate levels from additives such as sodium sulfate,^4^ azo dyes are widely used in the food, cosmetics, paper, pharmaceutical, and hair dye industries.^5^

Several methods have been reported in the literature as feasible alternatives for treating textile wastewaters. Biological treatments employing microbial consortia have shown strong potential for the degradation of organic pollutants in dye-contaminated wastewater. These systems replicate natural biodegradation cycles, reduce the need for chemical reagents, and minimize sludge generation as an additional waste to be treated.^6^ However, the toxicity of dyes and their intermediate byproducts can negatively affect microbial metabolism, inhibiting key enzymes such as azoreductases, laccases, and peroxidases, thereby limiting decolorization efficiency.^7,8^

Azo dyes feature one or more diazenyl (−N=N−) chromophore groups that connect aromatic (aryl) structures, conferring high chemical and photostability. Their bulky, rigid aromatic–azo configuration limits membrane permeability, making them resistant to cellular uptake and biodegradation.^9,10^ In addition, the anaerobic biodegradation of azo dyes frequently results in the formation of aromatic amines. These compounds are often more toxic than their precursor dyes, as their lower molecular weight and higher lipophilicity grant them greater cell membrane permeability. This facilitates their entry and interaction with intracellular components, which amplifies their cytotoxic and genotoxic effects.^11–13^

To overcome these limitations, advanced oxidation processes (AOPs) have emerged as a promising alternative.^14,15^ Among them, hydrodynamic cavitation (HC) is an efficient solution and can be used either individually or in combination with other oxidation processes.^16^ HC involves the formation, growth, and collapse of microbubbles created within cavities induced by spatial pressure variation, which occurs when the liquid passes through devices, such as venturi tubes or orifice plates. When microbubbles collapse, generating free radicals in “hotspots,” oxidation of the target compound occurs.^17,18^ The degradation of organic pollutants primarily involves two mechanisms: oxidation by hydroxyl free radicals (^•^OH), which preferably occurs with nonvolatile pollutants, and the thermal decomposition of compounds trapped within the cavity.^19^

Ozonation is widely recognized as a powerful AOP for the degradation of persistent organic pollutants. When ozone (O_3_) is dissolved in water, it acts through two complementary pathways: (i) direct oxidation, attacking azo bonds and aromatic rings; and (ii) indirect oxidation, in which O_3_ decomposes into highly reactive ^•^OH, enabling nonselective mineralization of complex organic structures.^20,21^ The efficiency of ozonation can be enhanced by combining it with HC, which produces extreme microenvironments with transient temperatures of >1000 °C and pressures exceeding 100 MPa. These conditions accelerate O_3_ decomposition inside the collapsing bubbles, thereby boosting the in situ production of ^•^OH and other reactive oxygen species.^22–24^ This acceleration of O_3_ activation by HC is the basis of the synergistic effect, leading to the faster elimination of stable intermediate byproducts.

Based on this scenario, this study evaluates the performance of individual and combined HC and O_3_ processes for the treatment of textile wastewater containing triazo dye. In addition, this work explores the integration of these AOPs as pre- and posttreatment stages to conventional biological systems, conditions rarely explored in the literature. The study provides novel evidence of synergy between cavitation-induced radicals and O_3_ decomposition in saline, dye-rich matrices, contributing mechanistic and scalability insights relevant to AOP integration.

## 2 Materials and Methods

### 2.1 Textile Wastewater Composition

The wastewater was formulated based on the composition of effluents generated from denim dyeing and washing processes in rural areas of Recife (Pernambuco, Brazil), serving as a model for textile effluents, as outlined by Amorim et al.^25^ It contained dye (65 mg L^–1^), ethanol (1.0 g-COD L^–1^), K_2_HPO_4_ (0.25 g L^–1^), NaCl (1.0 g L^–1^), NH_4_Cl (1.0 g L^–1^), and NaHCO_3_ (0.12 g L^–1^) as the alkalinity source. Micronutrients were supplied by adding an aliquot of 1 mL L^–1^ of a trace element solution, with the following composition (in mg L^–1^): nitriloacetic acid (12.8), FeCl_3_·6H_2_O (1.35), MnCl_2_·4H_2_O (0.1), CoCl_2_·6H_2_O (0.024), ZnCl_2_·4H_2_O (0.1), CuCl_2_·2H_2_O (0.025), H_3_BO_3_ (0.01), Na_2_MoO_4_·2H_2_O (0.024), Na_2_SeO_3_·5H_2_O (0.026), and NiCl_2_·6H_2_O (0.12).^26^ The dye used was Direct Blue 71 (DB71) (CAS 4399-55-7), purchased from Sigma-Aldrich, with a purity of 50%. Its molecular formula is C_40_H_23_N_7_Na_4_O_13_S_4_ (molecular weight of 1029.87 g mol^–1^), and it has a maximum absorption wavelength of 581 nm. Figure S1 shows its molecular structure.

### 2.2 Experimental Design

The study was divided into three experiments. The first one consisted of evaluating the effect of the AOP as a pretreatment for anaerobic digestion. The second experiment aimed to evaluate AOP as a posttreatment of the anaerobic effluent. Finally, the third experiment was performed to optimize the AOP parameters. The AOPs used in this study comprised HC and HC combined with ozone. This experimental configuration was previously described in Pereira et al.^27^ and is shown schematically in Figure S2.

During experiments I and II, influent and effluent samples from anaerobic bioreactors were subjected to the same operating conditions in AOP system, namely, pressure of 4.5 bar, a cavitation number of 0.33 (obtained from a flow rate of 0.045 m^3^ s^–1^ and a corresponding velocity of 25.71 m s^–1^), and an ozone flow rate of 1.0 g h^–1^. In experiment III, the ozone dosage and exposure time were varied, as further detailed in Section 2.2.3. O_3_ was produced from compressed oxygen (99.5%) using a myOZONE M10 generator (Brazil), as previously described by Pereira et al.^27^ The generator includes an integrated digital monitor displaying the O_3_ concentration, oxygen flow, power, and instantaneous O_3_ production rate. The oxygen supply system (10 L min^–1^ total flow rate) was equipped with flow and pressure regulators.

#### 2.2.1 Experiment I—Hydrodynamic Cavitation Coupled with Anaerobic Batch Reactors

The effluent generated from the HC process was subjected to anaerobic batch reactors. Two conditions were evaluated: with pretreatment (P) and with no pretreatment (NP). In the NP conditions, the textile wastewater was not subjected to the HC process. The performance assessment of the anaerobic bioreactors focused on methane production, organic matter removal, and dye decolorization. Both experiments were conducted in penicillin bottles with a total capacity of 100 mL. The working volume in each reactor was 80 mL, leaving a headspace of 20 mL at the start of the experiment.

The inoculum of each anaerobic bioreactor consisted of a 50:50% v/v mixture of biomass sourced from the Ideal LTDA poultry slaughterhouse and biomass from a continuous UASB reactor, which had been preadapted to the DB71 dye (Figure S3). The initial biomass concentration of 2.5 g of VSS L^–1^ was applied under all conditions. After the addition of centrifuged biomass and textile wastewater, the bioreactors were sealed with butyl rubber stoppers and metal crimp caps to ensure hermetic sealing. Two needles were attached to a three-way valve for separate collection of biogas and effluent. The bottles were purged with nitrogen gas for 10 min to ensure anaerobic conditions in the medium.^28^ Throughout the operational period, the reactors were maintained at 30 °C in a climate-controlled chamber.

The HC reactor consisted of a closed recirculation system with a total working volume of 2.4 L, including a stainless-steel tank, a heat exchanger to maintain the temperature at 25 ± 3 °C, a centrifugal pump (1.0 HP, KSB P1000TNG model) providing recirculation through an orifice plate cavitation device, and pressure gauges positioned upstream and downstream of the plate. The cavitation device was fabricated from stainless steel and consisted of a 5 mm thick plate with a single circular orifice of 1.5 mm diameter, as reported by Pereira et al.^27^ The treated liquid was continuously recirculated for 120 min during each test.

#### 2.2.2 Experiment II—Hydrodynamic Cavitation Coupled with Ozonation

The influent and effluent samples from the anaerobic bioreactor were subjected to an ozonation process combined with a HC system to evaluate the potential of O_3_ as an oxidizing agent for dye transformation and the feasibility of posttreatment for the biological process. Both samples were independently exposed to the same O_3_ load for 80 min, with samples collected at 20, 40, 60, and 80 min.

#### 2.2.3 Experiment III—Optimization of AOP Parameters

The optimization tests for AOP conditions were conducted according to a central composite design (CCD). The two independent factors were ozone flow rate and reaction time, resulting in *k* = 2 variables. The axial distance (α) was determined following the rotatable design criterion where α = (2^*k*^)^^(1/4)^^ corresponding to α = 1.41 for two factors. The final matrix consisted of 13 experiments: four factorial points (−1, +1), four axial points (−1.41, +1.41), and five replicates at the central point (0, 0) for estimation of experimental error and to improve model precision (Table S1). This methodology rigorously determines the experimental error through the inclusion of five repetitions of the central point and ensures robustness of the empirical model.

The experimental design, employing CCD to generate the response surface, was applied exclusively to the effluent from the anaerobic treatment. The response variable was the organic load removal, measured as the total organic carbon (TOC) removal efficiency (in %). The matrix presents the data processed using Chemoface 1.61 software provided in the Supporting Information (Table S2). The response surface was generated from the second-order empirical equation, based on the variables O_3_ flow rate and time, and their interaction, as presented in eq 1. *Y* is the predicted response (TOC removal); β_0_, β_1_, and β_2_ are the linear coefficients; β_11_ and β_22_ are the quadratic coefficients; *X*_1_ and *X*_2_ are the independent variables (O_3_ flow rate and time)

1 $$Y=\beta_{0}+\beta_{1}X_{1}+\beta_{2}X_{2}+\beta_{11}X_{1}^{2}+\beta_{22}X_{2}^{2}+X_{1}X_{2}$$

The optimization study focused on O_3_ dosage and treatment time under neutral pH, a temperature of 25 ± 3 °C, and operating pressure (4.5 bar), which were previously identified as optimal conditions for this reactor design.^27^ These parameters were kept constant to isolate the synergistic effect of HC + O_3_ and ensure the representativeness for real textile effluent matrices.

### 2.3 Analytical Methods and Data Analysis

Liquid samples from all experiments were analyzed according to the chemical oxygen demand (COD) and TOC following the Standard Methods (APHA, 2017).^29^ The biotransformation of DB71 and aromatic amine production were qualitatively assessed through UV–vis spectrophotometric measurements based on the methodology described by Pinheiro et al.^30^ In addition, decolorization efficiency (in %) was calculated using eq 2, in which *A*_feed_ is the absorbance of the feed; *A*_eff_ is the absorbance of the effluent; and *d*_λ_ is an infinitesimal wavelength interval.^31^ This procedure allowed the quantitative determination of color removal based on the integrated absorbance within the 400-700 nm wavelength range. UV–vis measurements were performed using baseline correction and 1 cm quartz cuvettes, with distilled water as a blank

2 $$decolorization\;\left(\%\right)=\left(1-\frac{\int_{400\;nm}^{700\;nm}A_{eff}\cdot d_{\lambda }}{\int_{400\;nm}^{700\;nm}A_{feed}\cdot d_{\lambda }}\right)\times 100$$

For the tests in anaerobic batch assays, the composition of headspace biogas was analyzed with a Shimadzu GC-2014 gas chromatograph equipped with a thermal conductivity detector (GC-TCD), employing hydrogen as the carrier gas at 24 mL min^–1^ with an HP Plot/Q column (30 m, 0.53 mm, 40 μm). The injector and detector temperatures were set at 160 and 170 °C, respectively. The oven temperature was initially maintained at 35 °C for 2 min and increased at 60 °C min^–1^ to 170 °C. The lower limits of quantification for CH_4_ and CO_2_ were 42.6 and 60.5 ppm_v_, respectively. The biogas composition analytical validation employed calibration gases of known composition to ensure precision and accuracy.^32^ The concentrations of methane (CH_4_) and carbon dioxide (CO_2_) were determined by calculating the moles of each gas from the chromatograph area. Internal pressure in each flask was measured using a pressure gauge (TPR-18 pressure transducer Desin Instruments) before sampling. This pressure, along with temperature (37 ± 2 °C) and headspace volume, allowed for the calculation of total moles in the reactor using the universal gas law. These tests were analyzed based on the accumulated methane measured over the operational time in normal liters (at *T* = 273.15 K and *P* = 1 atm) at a given time. The modified Gompertz equation^33^ was applied to the collected experimental data during the incubation phase. The kinetic equations and experimental procedures were carried out as described by Dias et al.^28^

For the statistical analysis of the data, a one-way analysis of variance (ANOVA) was applied using the PAST software (version 4.03). Significant differences were considered at a *p*-value ≤0.05, assuming a 95% confidence interval.^34^

## 3 Results and Discussion

### 3.1 Experiment 1: Hydrodynamic Cavitation Coupled with Anaerobic Batch Reactors

The effluent resulting from HC was subjected to a biological process in anaerobic batch conditions, P, and compared to the raw textile wastewater that was not subjected to the cavitation process, i.e., NP. In this context, biomethane production, organic matter removal, and dye decolorization were assessed. From the kinetic adjustment to the modified Gompertz model, the kinetic parameters (methane production potential and maximum methane production rate (*r*_max_)) were obtained, as presented in Table 1.

**Table 1:** Adjustment of the Kinetic Data to the Gompertz Model for the Conditions with Ethanol and Sludge

test	*P*_max_ (mL)	*r*_max_ (mL days^–1^)	λ (h)	*R* ^2^
no pretreatment (NP)	2.96 ± 0.09	0.16 ± 0.05	20.5 ± 2.7	0.96
pretreatment (P)	6.81 ± 4.29	0.14 ± 0.03	16.9 ± 4.8	0.92

When evaluating the kinetic data from the anaerobic batch reactors, it is observed that the methane production rate showed similar values under both conditions, 0.16 ± 0.05 and 0.14 ± 0.03 mL days^–1^ for the NP and P, respectively. Although the maximum volumetric methane production was 2.3 times higher in the P condition (*p* < 0.05), both conditions exhibited similar lag phases, 20 h for NP and 17 h for P, and the experimental data fitted the Gompertz model with a correlation coefficient (*R* ^2^) higher than 0.90. The graphs of accumulated methane production for the NP and P conditions can be seen in Figure 1.

**Figure 1:**
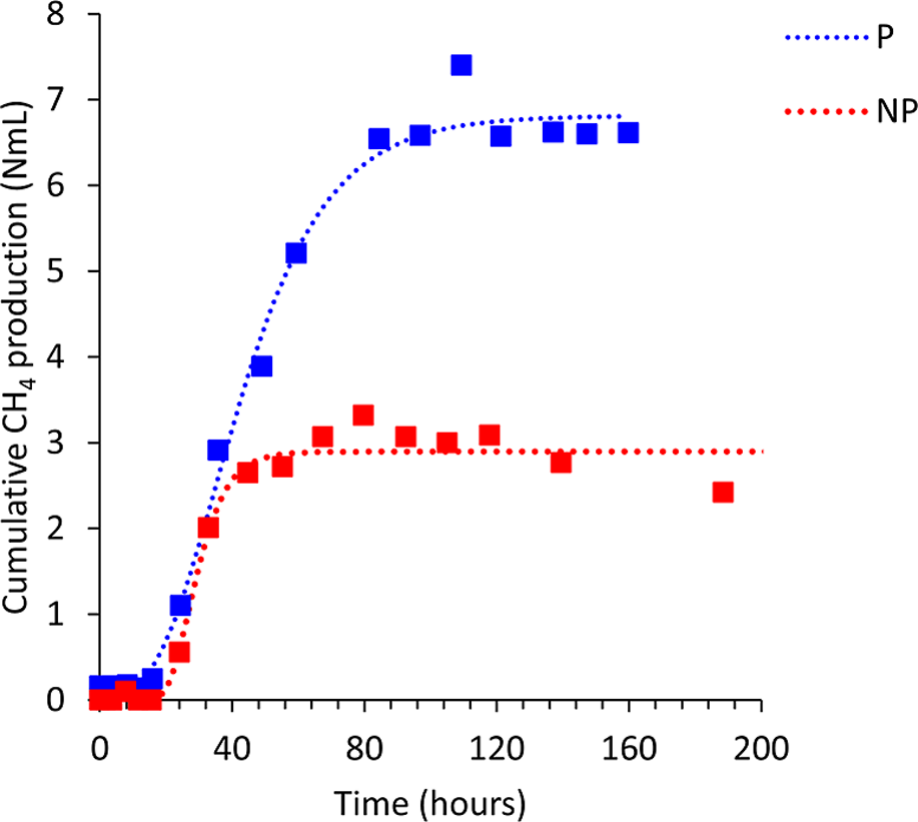
Accumulated methane gas production under conditions P and NP by cavitation during the operational period of anaerobic batch reactors.

The observed behavioral similarity in terms of the organic matter removal rate was also confirmed by the ethanol decay measured as COD (Figure 2). Despite the quantitative difference in the maximum accumulated methane gas production between the conditions, both exhibited similar overall efficiencies, with 88.3% for NP and 89.0% for P, and the statistical tests (one-way ANOVA) did not indicate a difference between them (*p* > 0.05). This is strong mechanistic evidence that the physical action of HC targeted the recalcitrant dye structure, inducing molecular destabilization and reducing its inhibitory effect on the methanogenic community, thereby facilitating the electron transfer pathways essential for bioenergy recovery.

**Figure 2:**
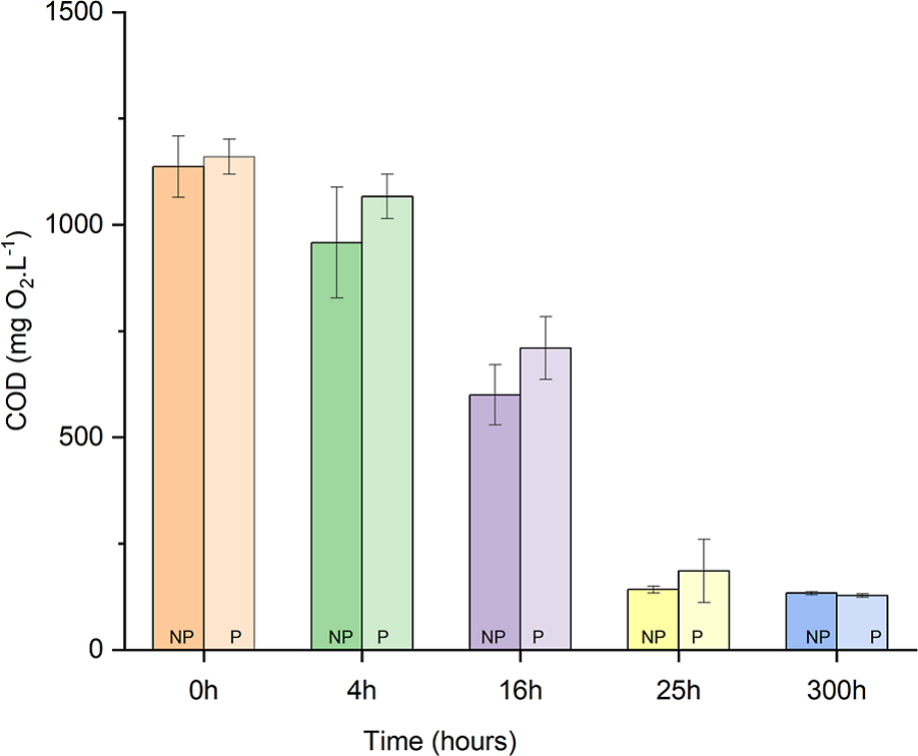
Graph of COD concentration in mg O_2_ L^–1^ under conditions NP and P by HC during the operational period of the anaerobic batch reactors, with samples collected at 0, 4, 16, 25, and 300 h.

In terms of dye removal, absorbances at a wavelength of 581 nm were evaluated according to Pinheiro et al.^30^ (Figure 3). The overall system efficiency was 95.7% for both the NP and P conditions. Although both conditions exhibited equal removals in overall terms and during the first 25 h, when approximately 84% of the dye had been reduced, during the first 16 h, the highest reduction in the dye peak occurred in the P condition, at 74.7%, compared to only 57.2% for the NP condition. It is observed that concurrently with the onset of methane production at approximately 17 h, the highest dye removal occurred for the P condition, while in the first 24 h, both conditions already exhibited volumetric methane production and similar dye removal efficiency.

**Figure 3:**
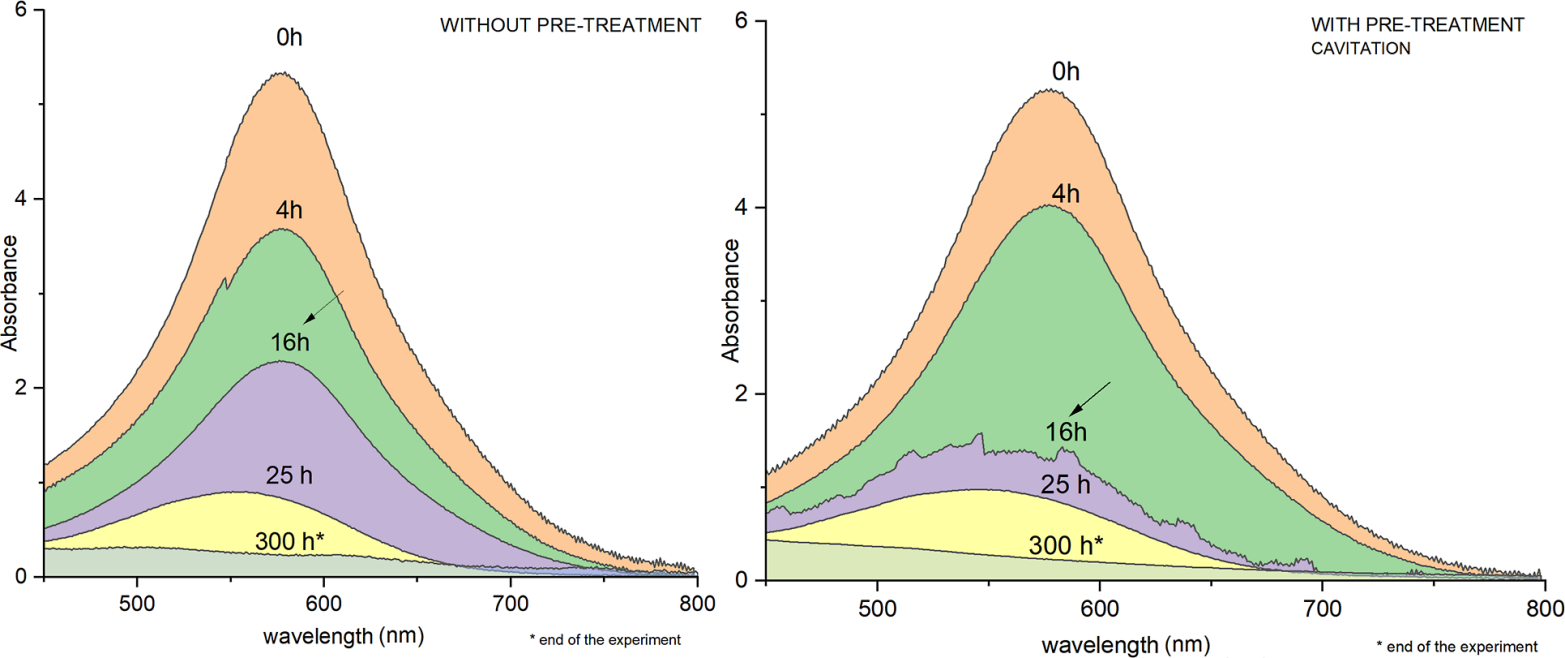
UV–vis spectrum between 450 and 800 nm for the conditions NP and P by cavitation during the operational period of the anaerobic batch reactors. The samples were collected at 0, 4, 16, 25, and 300 h. The maximum absorption wavelength of the dye is 581 nm. Absorbance readings were performed on samples diluted 5×. The plotted spectra represent values corrected for the dilution factor.

The scan presented for the P condition indicates that the exclusive HC process appears to have no significant effect on the direct cleavage of the azo bonds in the dye molecule (absorbance = 5.24) when compared to the NP condition, which shows the same peak absorbance (absorbance = 5.32). This evidence the efficiency and consolidation of biological processes in the removal of color originating from dyes.^31,35^ Despite a range of studies in the literature reporting that, although the efficiency of removing recalcitrant compounds using exclusive HC is low, this mechanism positively affects the biodegradability of these compounds, as determined by the biochemical oxygen demand/COD ratio, when used as a pretreatment preceding anaerobic biological processes.^36^

Gogate et al.^37^ studied HC as a pretreatment for domestic and industrial wastewaters before anaerobic treatment. They found that the hydraulic retention time for domestic sewage decreased from 60 to 21 h and organic matter removal improved, with COD removal increasing from 28.1% to 44.2% for industrial wastewater. For industrial cases, cavitation combined with Fenton’s reagent was essential. The authors attributed the benefits of HC pretreatment to reduced compound complexity and toxicity, promoting microbial growth.

HC can facilitate dye decolorization through the extreme localized temperatures and pressures generated by bubble collapse. However, the polarity of the molecules critically affects their susceptibility to cleavage. Hydrophobic dye molecules tend to concentrate at the collapsing bubble interface, where the intense energy release can cleave chromophore groups, leading to decolorization, whereas more polar molecules distribute in the bulk fluid and may be less efficiently degraded under such conditions.^38^

Askarniya et al.^39^ studied the dyes Tartrazine, Ponceau 4R, and Coomassie Brilliant Blue (CBB), comparing the efficiency of cavitation alone and with chemical agents. Decolorization occurred only at pressures above 3 bar, peaking at 6 bar, with CBB achieving the highest removal efficiency of 45.6%. The greater efficiency of CBB was due to its hydrophobic nature. DB71, similar to Ponceau 4R, showed a low removal efficiency with cavitation, explaining its poor decolorization.

To address this challenge, one option is to combine HC processes with oxidizing compounds that can assist in the transformation of these highly complex molecules. There are numerous reports on the association of chemical agents with the HC system, including photocatalysis, Fenton’s reagent, carbon tetrachloride, and ozonation.^18^ Soeira et al.^40^ also reported that by adding hydrogen peroxide as a chemical agent, there was an increase in the removal efficiency of the recalcitrant compound melanoidin from 14.2% to 60.3% using a HC system similar to that described in this work. In summary, HC does not directly cleave dye molecules but induces structural changes that reduce the molecular complexity and increase the biodegradability of the dye structure, facilitating its reduction in the anaerobic biological treatment. This partial destabilization reduces the inhibitory effects of azo dyes on the microbial community. Replicated batches demonstrated that HC pretreatment resulted in faster electron transfer kinetics, leading directly to the observed increase in methane production and the faster initial dye reduction (74.7% in 16 h for P vs 57.2% for NP). This establishes a clear mechanistic link between the physical pretreatment and the enhanced bioenergy recovery.

### 3.2 Experiment 2: Hydrodynamic Cavitation Coupled to Ozonation (HC + O_3_)

Figure 4 shows the results of the AOP tests with the influent and effluent samples from biological treatment of textile wastewater. The influent sample subjected to the combined HC + O_3_ showed a rapid decrease in absorbance (581 nm) from 7.49 to 0.06 over the total duration of 80 min, resulting in a color removal efficiency of 99.2%. While initial decolorization is rapid, the true synergistic benefit of HC lies in the accelerated degradation of intermediate compounds. It was observed that nearly all of the dye was removed within the first 20 min with the applied O_3_ dosage. The overlap observed in the scans of the influent samples taken at 20, 40, 60, and 80 nm corroborates this observation. The spectrophotometric scanning in the wavelength range of 200–800 nm and the photographs of the analyzed samples for the influent sample are presented in Figure 4a. Based on the scans between the wavelengths of 200–500 nm, there was no evidence of the formation of byproducts or of color recovery in the sample, as observed in the biological treatment. This rapid, complete suppression of UV–vis absorption in this wavelength range strongly suggests the efficient oxidative cleavage of aromatic amines and other UV-absorbing, potentially toxic, intermediate byproducts formed after the azo bond cleavage.

**Figure 4:**
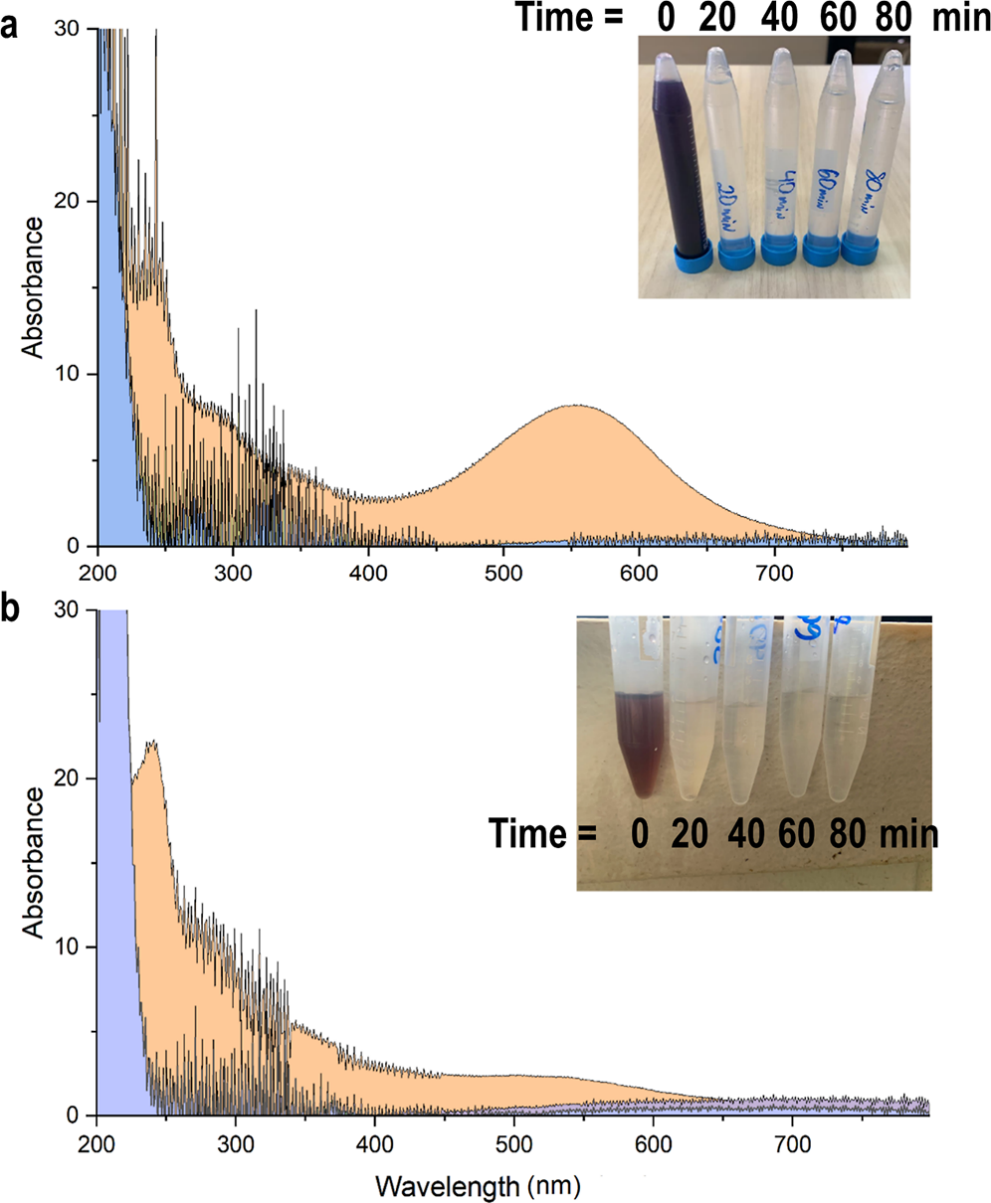
UV–vis spectrum of influent (a) and effluent (b) samples at times 0, 20, 40, 60, and 80 min. The scans from 20 to 80 min overlap (bluish-colored areas). Absorbance readings were performed on samples diluted 5×. The plotted spectra represent values corrected for the dilution factor.

The mechanism of dye degradation in the hybrid HC + O_3_ process involves both direct and indirect oxidation routes. In the direct pathway, O_3_ acts as an electrophilic oxidant that reacts with electron-dense sites in the dye structure, particularly the azo (−N=N−) bonds and aromatic rings, causing chromophore cleavage and color removal. The indirect mechanism arises from the decomposition of O_3_ into reactive oxygen species, primarily ^•^OH radicals.^41,42^ The HC process accelerates O_3_ decomposition by generating microbubble collapses that create extreme local conditions (*T* > 1000 °C, *P* > 100 MPa), enhancing ^•^OH formation.^43,44^ These radicals then promote nonselective oxidation of intermediate aromatic amines, contributing to the overall mineralization of DB71. The synergistic action of HC and ozonation thus ensures simultaneous chromophore cleavage and the advanced oxidation of degradation intermediates.

Wang et al.^42^ also reported the success of combining the HC process with O_3_. The authors stated that the dye removal, measured by UV at 254 nm and through the evaluation of macromolecular compounds containing C=C and C=O double bonds, indicated that both the dye and its byproducts were effectively degraded, with a 90% reduction in color within 30 min of experimentation. The authors also noted that HC alone, without other oxidants, would not be sufficient to fully degrade these compounds, as observed by comparing the two conditions.

The influent wastewater matrix contained ethanol (added at 1.0 g-COD L^–1^), which is known to act as a potent ^•^OH radical scavenger.^45^ The fact that high decolorization was achieved (Figure 4a) demonstrates the high resilience of the HC + O_3_ system to potential scavengers. This high AOP efficiency is maintained because the powerful local generation of radicals within the cavitation hotspots is sufficient to overcome the scavenging competition from the bulk solution. Furthermore, pollutants with moderate hydrophobicity, such as the DB71 dye, may preferentially migrate to the bubble interface, gaining a kinetic advantage over hydrophilic scavengers like ethanol. In addition, the salts in the wastewater (NH_4_Cl, NaCl, NaHCO_3,_ and K_2_HPO_4_) can also influence O_3_ activation and radical stability. Bicarbonate and phosphate ions act as mild ^•^OH scavengers, thereby reducing the contribution of radical-driven oxidation,^46^ while chloride ions may react with ^•^OH to generate chlorine-based radicals of lower oxidation potential.^47^ Nevertheless, the continuous cavitation events inherent to the HC + O_3_ process sustain O_3_ decomposition, mitigating the inhibitory influence of both organic and inorganic scavengers.

The effluent from the anaerobic system was subjected to the same O_3_ dosage as for the influent sample. The samples analyzed within the specified time intervals showed a decrease in the residual absorbance in the anaerobic effluent from 1.76 to 0.03, resulting in a 98.4% dye removal. Replicating the same behavior observed with the influent sample, it was noted that the first 20 min were sufficient to remove the maximum amount of the remaining dye, with peak overlap observed. The peaks observed in the sample at time 0, with emphasis on the highest peak at a wavelength of 242 nm, also showed a reduction (Figure 4b).

Thus, it can be concluded that ozone in this experiment was effective in removing the analyzed compounds. Mella et al.^48^ evaluated the combination of chemical and physical processes for the removal of Acid Red 357 dye. The authors used 1 g-O_3_ h^–1^ and achieved a dye removal of 85.4%. They attributed ozone’s ability to break double bonds, such as those in azo dyes (−N=N–, Figure S1), as well as its broad capacity to break unsaturated bonds in aromatic compounds present in humic substances and chromophore groups.

When the samples were evaluated in terms of TOC removal, efficiencies of 48% in the influent and 45% in the effluent were observed (Figure S4). It is worth noting that the influent showed a greater reduction in TOC during the first 20 min, with a 43% decrease, and remained stable in subsequent collections at 40, 60, and 80 min. In contrast, in the effluent sample was removed only 26% of TOC in the first 20 min, followed by an additional 10% over the next 20 min, reaching stability only after 40 min. In the first 20 min, it is possible to oxidize at least 43% of the dye molecule in the influent, while in the effluent, even though the scan indicates that the remaining dye was removed in the same time interval, organic molecules from the anaerobic treatment and resulting from the oxidation of the remaining dye molecule were still solubilized and capable of being oxidized. This occurs only after 60 min, when the removal of organic matter stabilizes, indicating that what could be oxidized under those conditions had already been oxidized. This analysis also reveals that not all the organic load is removed from the influent and effluent samples, even during the 80 min of system operation.

Punzi et al.^49^ combined an anaerobic biofilm reactor with ozone posttreatment, testing doses of 0.13, 0.26, and 0.52 g-O_3_ L^–1^. They noted reductions in Remazol Red dye peaks (600 nm) and aromatic amines (200–400 nm), indicating the effective cleavage of the dye and the opening of aromatic rings. TOC analysis revealed mineralization removals of 31%, 13%, and 12% after 4 min at the maximum ozone dosage for dye concentrations of 100, 500, and 1000 mg L^–1^, respectively. However, the authors highlighted limitations in treating high dye concentrations such as 1 g L^–1^.

Based on the analysis conducted in this experiment, it was observed that O_3_ was effective in removing the dye and its byproducts. To mitigate the disadvantages of exclusive ozone use, such as high costs, the combination of ozone with HC can be employed, for example, to create more economic and effective systems for the treatment of recalcitrant compounds.^18^

Recent trends in AOPs focus on intensified radical generation methods, such as the use of O_3_ nanobubble technology for enhanced mass transfer and radical production, which has shown success in degrading persistent pollutants like tetracycline.^50^ Similarly, the application of percarbonates (e.g., activated sodium percarbonate, SPC) in hybrid systems like SPC/O_3_/HC offers a promising chemical route for activating oxidants via cavitation for compounds such as 1,4-dioxane.^51^ Our study focuses on the physical–chemical synergy of HC/O_3_, demonstrating an efficient, reagentless activation pathway that avoids the recurring cost of external chemical oxidants. Moreover, this work extends the conceptual framework of cavitation-assisted oxidation by demonstrating that mechanical turbulence can catalyze the decomposition of O_3_ in textile wastewaters. The HC/O_3_ system presents a distinct advantage over other hybrid ozonation processes, such as UV/O_3_ and TiO_2_/O_3_,^52^ as it promotes in situ radical generation without the need for external radiation or catalyst addition.

### 3.3 Experiment 3: Optimization of AOPs Parameters

In the HC process, variations in parameters such as the O_3_ dosage applied, the exposure time of the wastewater to the chemical agent, the pressure used in the experiment, and the cavitation number can directly affect the results of the processes in terms of the removal efficiency of recalcitrant compounds.^53–55^ To estimate the threshold value that provides process efficiency under optimal conditions, the curve associating the dosage of the O_3_ with exposure time was analyzed from the response surface. In the experiments conducted for the experimental design, out of the 13 experiments performed, the efficiency of organic matter removal in terms of TOC ranged from 7.6% to 58.3%. The minimum and maximum initial values for ozone dosage were 1.5 and 4.5 g-O_3_ h^–1^, respectively, and the exposure time varied from 30 to 100 min. The concentration of TOC in the initial sample before being subjected to the defined dosages of O_3_ in conjunction with HC was 58 mg L^–1^. The data comprising the resulting matrix of the CCD are presented in Table S2.

As the dosage was increased and the exposure period was extended, an increase in the removal efficiency of the substances contributing to TOC (e.g., dye, byproducts, and some residual ethanol) was observed. Thus, these variables had a significant impact on the removal of this molecule. This analysis is supported by the Pareto diagram presented in Figure S5, which indicates the effect of the variables presented on the removal efficiency. The graph shows that the variable time (*X*_2_) is more significant in relation to TOC removal than the variable dosage of O_3_ (*X*_1_) and even the interaction between both variables *X*_1_*X_2_. The significance values of the variables and their interaction, also obtained from the Chemoface software, are presented in Table 2. The fitting of the second-order empirical model to the experimental data demonstrated high statistical rigor. As detailed in Table S3, the resulting quadratic model showed a coefficient of determination (*R* ^2^) of 0.99. This value confirms that the empirical model is highly reliable, validating the adequacy of CCD for process optimization.

**Table 2:** Summary of Numerical Values Related to the Effects of the Variables O_3_ Flow Rate (*X*_1_), time (*X*_2_), and Their Interaction (*X*_1_ *X_2_)

parameter	effect	error	*t*	*p* value	significance
O_3_ flow rate (*X*_1_)	16.5200	1.8948	8.7188	9.5315 × 10^–4^	yes
time (*X*_2_)	20.5423	1.8948	10.8417	4.107 × 10^–4^	yes
*X*_1_*X_2_	–16.3250	2.6796	–6.0924	0.0037	yes

Based on the presented data, the response surface was obtained using the quadratic model derived from eq 3. The optimal range observed was with an O_3_ dosage of 3 g h^–1^ at 65 min, resulting in an efficiency of 56.2%. The response surface is shown in Figure 5. It was observed that at lower ozone dosages, specifically 0.87 g-O_3_ h^–1^ with exposure times less than 86.5 min, the model yields negative efficiency values, which are not reproducible in practice due to the nature of performance efficiency. As the dosage and time increase, the efficiency also increases, reaching a maximum of 58.3% at a dosage of 3 g h^–1^ and an exposure time of 114.5 min. Beyond this point, increasing the dosage leads to a decrease in efficiency or it becomes necessary to reduce the exposure time. At the optimized HC + O_3_ condition, the specific oxidant demand was approximately 40 g-O_3_ g^–1^ TOC removed (56% efficiency).

3 $$Y=52.15+8.26\cdot X_{1}+10.27\cdot X_{2}-14.38\cdot X_{1}^{2}-3.95\cdot X_{2}^{2}-8.16\cdot X_{1}X_{2}$$

**Figure 5:**
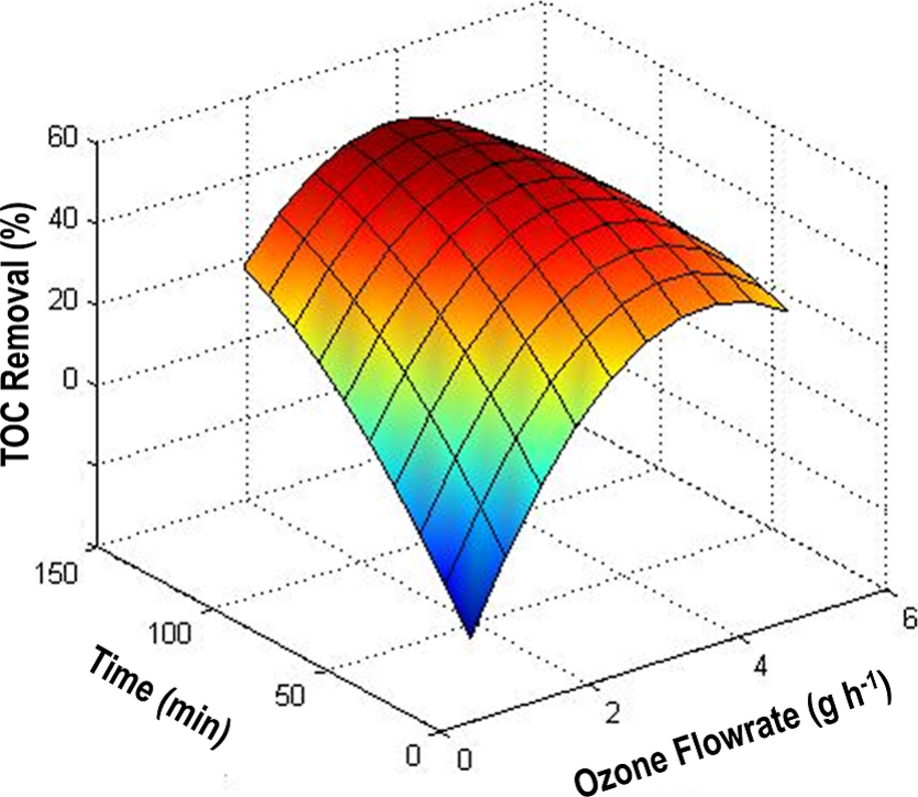
Graphical representations of 3D response surface plots of the model showing TOC removal as a function of time and O_3_ flow rate.

In the assays presented that comprised the response surface, analyses were performed at each point represented by the O_3_ dosage and exposure time to which the effluent samples were subjected. In addition to the TOC removal efficiency, spectrophotometric scan analyses were also evaluated in the collected samples. As in the previous experiment, these analyses were conducted to monitor the reduction of the remaining dye from the anaerobic effluent and the mineralization of the byproducts formed during this process. In the scan presented in Figure 6, it was possible to observe the decrease in the dye peak when comparing the sample subjected to 4.5 g-O_3_ h^–1^ for 100 min with the sample at time 0, which is the effluent from the anaerobic system that was not exposed to ozonation. The decrease in the dye peak was observed to be nearly complete. When analyzing the sample subjected to 1.5 g-O_3_ h^–1^ for 30 min, a reduction of 91.1% in terms of absorbance was noted.

**Figure 6:**
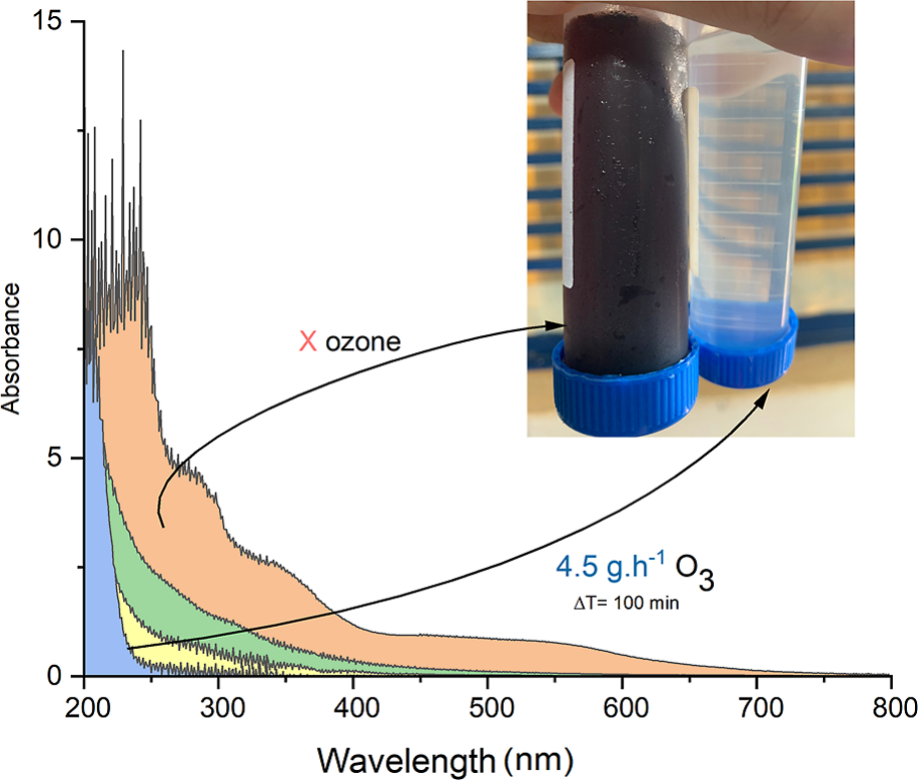
Spectrophotometric scan of the effluent sample: (orange) sample 0—without ozonation, (green) sample 1—1.5 g-O_3_ h^–1^, Δ*t* = 30 min, (yellow) sample 3—4.5 g-O_3_ h^–1^, Δ*t* = 30 min, and (blue) sample 4—4.5 g-O_3_ h^–1^, Δ*t* = 100 min. Absorbance readings were performed on samples diluted 5×. The plotted spectra represent values corrected for the dilution factor.

The TOC removal achieved in this study (maximum 58.3%) lies within the range typically reported for cavitation-assisted O_3_ processes treating real dye-bearing wastewaters. For example, Rajoriya et al.^56^ reported TOC reductions of around 48% after 120 min for HC coupled with O_3_ on textile-dyeing wastewater, highlighting that mineralization generally lags behind decolorization in complex matrices. In a broader survey of hybrid cavitation AOPs, Thanekar and Gogate^57^ compiled TOC removals that commonly fall in the 40–70% range for real wastewaters (with higher values occasionally observed in simplified model solutions), reinforcing the matrix dependence of mineralization. Gujar and Gogate,^58^ working with commercial dye industry effluents, reported moderate TOC decreases (typically ∼40–55% within 90–120 min) using cavitation-based hybrids, attributing the ceiling on mineralization to the presence of refractory organics and radical scavengers in saline, high-ionic-strength streams.

### 3.4 Scalability Implications and Operational Cost Analysis

The bench-scale experiments were critical for establishing the proof-of-concept and determining the optimal operational regime (optimized via CCD) for the polishing treatment of the biological effluent. The hybrid HC + O_3_ system employs technologies already consolidated in the industry (venturi-type reactors and ozone generators), which makes the technology inherently scalable. The CCD identified the optimal operational window of 3.0 g-O_3_ h^–1^ for 65 min to achieve 56% of TOC removal. These quantified parameters allow process engineers to accurately size the necessary O_3_ generation capacity and determine the necessary hydraulic retention time for specific industrial flow rates, providing the foundation for pilot-scale design.

Under the optimized condition (3.0 g-O_3_ h^–1^ for 65 min), the electrical energy per order (EEO) was calculated according to eq 4, where *P* (kW) is the HC and O_3_ power input during the HC/O_3_ process, *V* (L) is the volume of wastewater treated, *t* (h) is the reaction time, and *C*_i_ and *C*_f_ (mg L^–1^) are the initial and final TOC concentrations, respectively.^41^ Using a total power input of 0.9 kW (0.15 kW of an O_3_ generator +0.75 kW of a pump), *t* = 1.08 h, *V* = 2.4 L, and *C*_i_/*C*_f_ = 2.27 (corresponding to 56% TOC removal), the EEO was estimated at 19.0 kWh m^–3^ order^–1^. This value indicates a highly energy-efficient performance, especially when compared with other hybrid cavitation–ozonation systems. For instance, Choi et al.^59^ reported an EEO of 58.7 kWh m^–3^ order^–1^ for a hybrid HC/O_3_ system treating oxalic acid

4 $$EEO=\frac{P\times t\times 1000}{V\times 60\log\left(\frac{C_{i}}{C_{f}}\right)}$$

AOPs are known to be energy-intensive with electrical consumption representing the dominant operational cost, primarily associated with the HC pump and the O_3_ generation unit. However, the cost is justified by the critical performance gains that cannot be achieved with biological treatment alone: (i) the HC + O_3_ process rapidly achieves complete decolorization and eliminates toxic UV-absorbing byproducts in under 20 min; (ii) the system ensures the mineralization of recalcitrant compounds (∼56% of TOC removal in the optimal condition). Furthermore, the operational cost of the final AOP stage is mitigated by the bioenergy offset achieved upstream. The HC pretreatment significantly enhanced methane recovery (*P*_max_ was doubled), establishing a dual-benefit system that trades a higher energy input in the final stage for superior effluent quality and improved upstream bioenergy generation.

### 3.5 Limitations and Future Perspectives

It is acknowledged that the TOC removal <60% achieved by the optimal HC + O_3_ condition indicates incomplete mineralization, which is a trade-off accepted for the economic viability of the AOP. Future studies should focus on implementing sequential biological treatments (e.g., aerobic posttreatment) to handle the remaining organic load and achieve full mineralization. Furthermore, while spectral analysis provided strong evidence of the elimination of UV-absorbing toxic intermediates (e.g., aromatic amines), future work should employ advanced analytical methods (such as liquid chromatography coupled to high-resolution mass spectrometry) to precisely identify and quantify degradation products and perform standardized toxicity assays to fully validate the detoxification effect of the combined HC + O_3_ process.

Another important limitation concerns the use of synthetic wastewater containing a single commercial dye (DB71) rather than a real industrial effluent. Although the synthetic matrix was designed to replicate the physicochemical characteristics of real denim dyeing and washing wastewaters from Recife (Pernambuco, Brazil), it does not fully capture the variability and complexity of actual textile discharges. This controlled setup, however, ensured experimental reproducibility and facilitated the mechanistic assessment of each treatment stage (HC, O_3_, and anaerobic digestion). Future studies should extend the evaluation to real textile effluents containing mixed dyes and auxiliary chemicals to confirm the robustness and scalability of the hybrid HC + O_3_ process under practical operational conditions.

## 4 Conclusions

This study demonstrated the potential of HC and its synergistic combination with ozonation (HC + O_3_) for the treatment of textile wastewater containing the recalcitrant triazo dye DB71. HC alone did not significantly promote direct cleavage of azo bonds but improved wastewater biodegradability, resulting in faster electron transfer and enhanced methane production during subsequent anaerobic digestion. This highlights its value as a pretreatment step to support biological processes. In contrast, the combined HC + O_3_ system proved highly efficient, achieving rapid and near-complete dye decolorization (>99% within 20 min).

The integration of HC with O_3_ promoted free radical generation and led to the rapid elimination of UV-absorbing, potentially recalcitrant byproducts by 20 min, which represents the true synergistic effect and enhanced mineralization rate, overcoming the limitations observed when applying either process individually. This integrated approach is promising because it combines the advantages of advanced oxidation with biological processes, enabling more effective decolorization, higher biodegradability, and improved energy recovery through methane production (*P*_max_ doubled). While validation at the pilot scale is necessary, the defined operational window (3 g-O_3_ h^–1^ in 65 min via CCD) provides the essential engineering data for scale-up design. This work extends the conceptual framework of cavitation-assisted oxidation by demonstrating that mechanical turbulence can catalyze decomposition of O_3_ in dye-rich matrices typical of textile wastewaters, a condition rarely explored in prior research.

## Supplementary Material



## Notes

Detailed descriptions of the HC reactor setup and schematic representation; characteristics of the anaerobic inoculum; TOC data; CCD matrices; and the full ANOVA results for the quadratic regression model (PDF)
