# Supplementary material for: Advanced Oxidation via Hydrodynamic Cavitation and Ozonation for Enhanced Decolorization and Biodegradability of Triazo Dyes in Textile Wastewater
Source: ACS Omega. 2025 Dec 9;10(50):62067–77. doi: 10.1021/acsomega.5c09276 (PMC12750273; doi:10.1021/acsomega.5c09276)
Supplement: Supplementary file 1 [file ao5c09276_si_001.pdf]

Supporting Information

Advanced oxidation via hydrodynamic cavitation and ozonation for enhanced decolorization and biodegradability of triazo dyes in textile wastewater

Rhayssa de Brito[a]; Rodrigo B. Carneiro[a,b]*; Julio C. S. I. Gonçalves[c]; Sávia Gavazza[d]; Márcia H. R. Z. Damianovic[a]

[a] Biological Processes Laboratory (LPB), São Carlos School of Engineering, University of São Paulo (USP), 1100, João Dagnone Ave., Santa Angelina, 13563-120, São Carlos, São Paulo, Brazil.

[b] Laboratory of Chromatography (CROMA), São Carlos Institute of Chemistry, University of São Paulo (USP), 400, Trabalhador São-Carlense Ave., São Carlos, São Paulo, 13566-590, Brazil

[c] Institute of Technological and Exact Sciences, Federal University of Triângulo Mineiro (UFTM), 1400, Randolfo Borges Júnior Ave., Uberaba, Minas Gerais, 38064-200, Brazil

[d] Laboratory of Environmental Sanitation, Department of Civil and Environmental Engineering, Federal University of Pernambuco (UFPE), Acadêmico Hélio Ramos Ave., Recife, Pernambuco, 50740-530, Brazil

*Corresponding author - Tel: +55 (16) 3373-8358; ORCID: 0000-0002-9188-4133; rodrigocarneiro@sc.usp.br / rodrigobrazcarneiro@yahoo.com.br

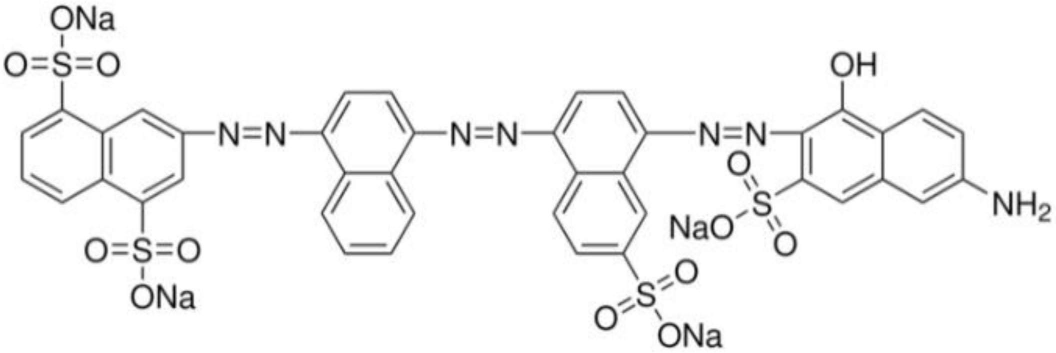

Figura S1. 1Molecular structure of the dye DB71

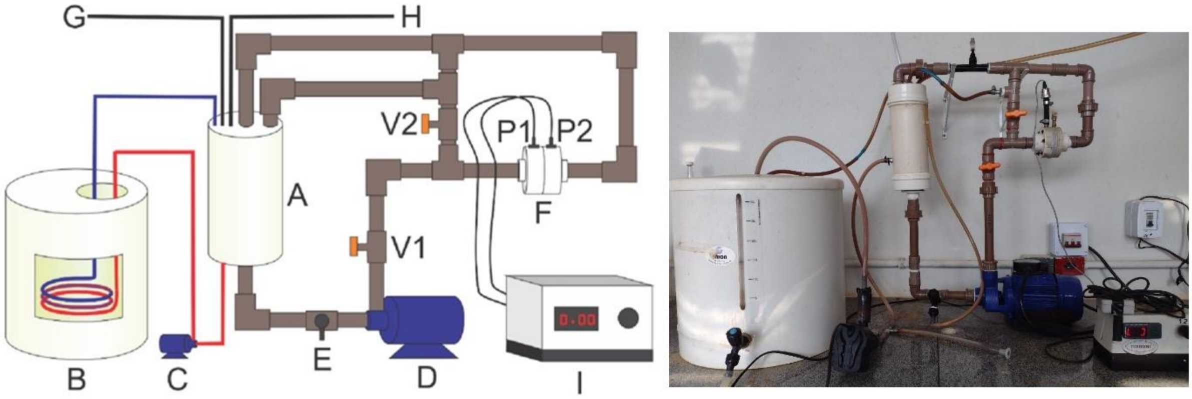

Figure S2. Schematic representation of the HC reactor set-up: **A** tank; **B** heat exchanger; **C** heat exchanger water pump; **D** peripheral hydraulic pump; **E** register for taking samples; **F** orifice plate; **G** O_3 inlet; **H** output of residual O_3; and **I** pressure gauge. Adapted from (Pereira et al., 2023).

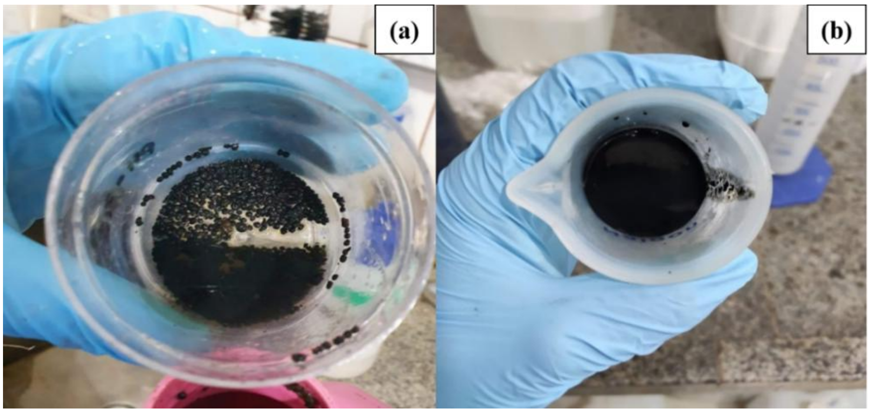

Figure S3. Sludge from the methanogenic inoculum from Ideal LTDA poultry slaughterhouse (a) and adapted to DB71 dye (b)

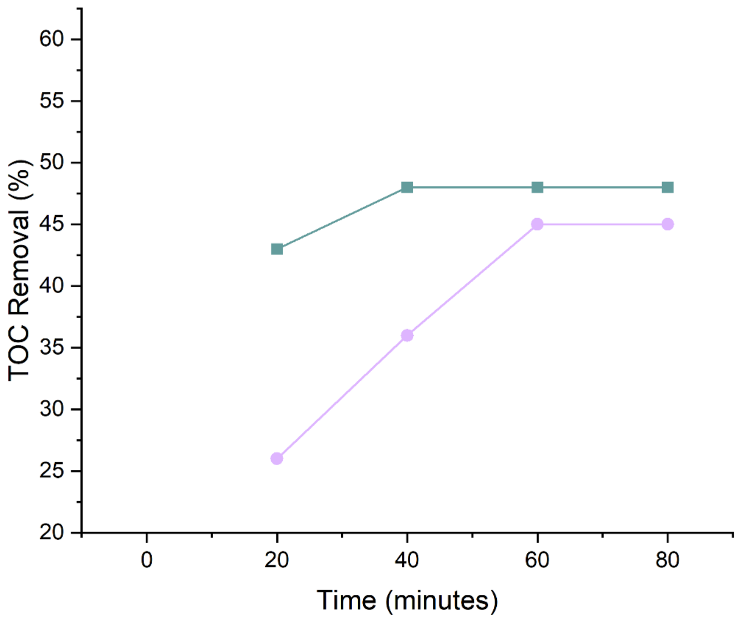

Figure S4. Graph of organic matter removal in terms of TOC for the influent (■) and the effluent (●) samples

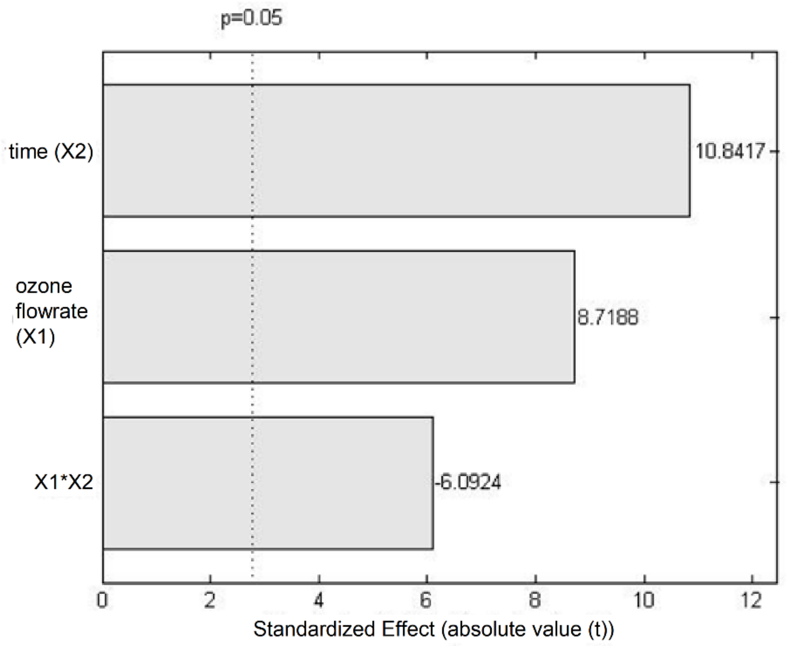

Figure S5. Pareto Chart of the standardized effect estimation for the dependent variables and observed and predicted values for the dependent variable ratio.

Table S1. Matrix of the actual values and coded values (in parentheses) obtained from Chemoface 1.61 software.

Assay	O_3 flowrate ($g\ h^{-1}$)	Time (minutes)
1	1.5 (-1)	30 (-1)
2	1.5(-1)	100 (1)
3	4.5 (1)	30 (-1)
4	4.5 (1)	100 (1)
5	0.8787 (-1.41)	65 (0)
6	5.1213 (1.41)	65 (0)
7	3 (0)	15.5 (-1.41)
8	3 (0)	114.49 (1.41)
9	3 (0)	65 (0)
10	3 (0)	65 (0)
11	3 (0)	65 (0)
12	3 (0)	65 (0)
13	3 (0)	65 (0)

Table S2. CCD matrix from different ozone flowrates (X1) and time (X2), with total organic carbon (TOC) removal efficiency as the response variable (Y).

Triplicates assays	Independent Variables (X)		Response Variable (Y)	
	Ozone flowrate X1 (g h^{-1})	Time X2 (minutes)	TOC (mg L^{-1})	TOC Removal (%)
1	1.5	30	53.17	7.63
2	1.5	100	32.13	44.18
3	4.5	30	33.86	41.17
4	4.5	100	31.62	45.07
5	0.89	65	50.95	11.48
6	5.12	65	38.07	33.86
7	3	15.50	40.99	28.79
8	3	114.49	24.01	58.29
9	3	65	25.22	56.18
10	3	65	27.51	52.21
11	3	65	29.4	48.92
12	3	65	28.32	50.80
13	3	65	27.27	52.62

Table S3. Summary of the analysis of variance results of the regression model for the TOC removal.

Parameter	Sum of Squares	Degrees of freedom	Mean Square
Regression	3126.5	5	625.2978
Residual	33.8502	7	4.8357
Lack-of-fit	5.1295	3	1.7098
Pure Error	28.7207	4	7.1802
Total	3160.3	12	
R^2	0.9893		
Adjusted R^2	0.9816		
